# Basic Psychological Need Profiles and Correlates in Physical Activity Participation: A Person-Centered Approach

**DOI:** 10.3389/fpsyg.2021.675639

**Published:** 2021-05-31

**Authors:** Chunxiao Li, Chee Keng John Wang, Koon Teck Koh, Kwang San Steven Tan, Shern Meng Tan, Wee Boon Ang, Liang Han Wong, Huat Neo Connie Yeo

**Affiliations:** ^1^School of Physical Education and Sports Science, South China Normal University, Guangzhou, China; ^2^National Institute of Education, Nanyang Technological University, Singapore, Singapore; ^3^Physical, Sports and Outdoor Education Branch, Ministry of Education, Singapore, Singapore

**Keywords:** need satisfaction, physical exercise, latent profile analysis, students, wellbeing

## Abstract

Guided by Basic Psychological Need Theory, we investigated the combined associations between need satisfaction and need frustration (*i*.*e*., need profiles) and their relations with theoretically relevant correlates including mindfulness, physical literacy, physical activity enjoyment, and physical activity. The participants were Singapore-based school students (*N* = 844, *M*_age_ = 12.45, SD_age_ = 1.99, boys = 53.1%) who completed a cross-sectional survey. The results of the latent profile analysis identified four distinct need profiles: profile 1–average satisfaction and frustration (*n* = 364, 44.1%); profile 2–low satisfaction (*n* = 251, 29.7%), above average frustration; profile 3–very high satisfaction, very low frustration (*n* = 144, 17.1%); and profile 4–high satisfaction, very high frustration (*n* = 85, 10.1%). Among these, profile 3 was the most adaptive one; it had the highest levels of mindfulness, physical literacy, physical activity enjoyment, and moderate-to-vigorous physical activity. Experiences of need satisfaction countered the negative effects of need frustration on these correlates. These findings enhance our understanding of students’ psychological need experiences and highlight the need for investigating the combined associations between need satisfaction and need frustration.

## Introduction

Basic Psychological Need Theory (BPNT; Deci and Ryan, 2000), one of the mini-theories in Self-Determination Theory, has been widely used for understanding human functioning (Vansteenkiste et al., 2020). BPNT posits that humans will achieve optimal functioning and well-being *via* the satisfaction of three basic psychological needs. By contrast, frustration of the three psychological needs will lead to human dysfunction and ill-being (Vansteenkiste and Ryan, 2013; Vasconcellos et al., 2019). Although these tenets are widely supported by a wealth of empirical evidence derived from variable-based analyses such as multiple regression, little research has examined the combined associations between need satisfaction and need frustration (*i*.*e*., need profiles) and their relations to human functioning (Rouse et al., 2020; Vansteenkiste et al., 2020; Warburton et al., 2020). To address this literature gap, we used person-centered analyses to examine need profiles and their associations with selected theoretically relevant correlates including mindfulness, physical literacy, physical activity (PA) enjoyment, and PA in school students.

Basic Psychological Need Theory posits that humans universally possess three basic psychological needs, which are the needs for autonomy, competence, and relatedness (Deci and Ryan, 2000). Humans satisfy their need for autonomy when they experience control over tasks, while the need for competence is satisfied when humans feel effective and capable of completing valued tasks. Lastly, the need for relatedness is satisfied when humans experience closeness and connection with significant others. According to BPNT, satisfaction of the three basic psychological needs (*i*.*e*., “bright side”) is essential for growth and positive development of humans. Recent advancements in BPNT have specified a “dark side”, in which the three basic psychological needs are actively frustrated or thwarted (Bartholomew et al., 2011). Hence, it is important to differentiate the difference between low need satisfaction and need frustration (Bartholomew et al., 2011; De Francisco et al., 2018). In the latter case, the three basic psychological needs are frustrated when humans are asked to do tasks against their will, experience a sense of failure and low confidence, and experience feeling excluded and rejected by others. Frustration of these needs are found to ensue dysfunction and ill-being, such as low levels of motivation and mental problems (Deci and Ryan, 2000; Vansteenkiste and Ryan, 2013; De Francisco et al., 2018). Furthermore, in comparison to low need satisfaction, need frustration is considered a better predictor of negative outcomes such as burnout, depression, and sedentary time, supporting the different roles of need satisfaction and need frustration in understanding human functioning (Bartholomew et al., 2011; Vansteenkiste et al., 2020; Warburton et al., 2020).

The establishment of the distinct concept of need frustration has spurred research to simultaneously examine need satisfaction and need frustration. However, most of the research so far have only explored this through variable-centered approaches such as multiple regression analysis (Warburton et al., 2020). Although the use of variable-centered approaches can shed light on explaining the relationships between a set of basic psychological needs and related variables, they provide limited information on the combined associations between need satisfaction and need frustration (Rouse et al., 2020). In other words, it is difficult to answer research questions such as “How are need satisfaction and need frustration combined to predict outcomes through the variable-centered approach?” As a supplement to the variable-centered approach, researchers have also employed person-centered approaches such as cluster analysis and latent profile analysis (LPA), focusing on identifying subgroups within a heterogeneous sample based on the shared similarities on a set of variables (Howard and Hoffman, 2018). Specifically, by employing the person-centered approach, we are able to identify subgroups with different combinations of need satisfaction and need frustration scores (*i*.*e*., need profiles) rather than “slicing” the participants into different need-related dimensions.

Indeed one proposition of BPNT is that there is an asymmetrical relationship between need satisfaction and need frustration (Vansteenkiste and Ryan, 2013), that is, while the presence of low need satisfaction does not imply the presence of need frustration, the presence of high need frustration necessitates the existence of low need satisfaction. There is, however, little evidence to support this proposition, as most of the studies conducted to identify need profiles are based only on need satisfaction scores (Vansteenkiste et al., 2020). Two recent studies examined need profiles based on need satisfaction and need frustration scores in the contexts of work, sport, and physical education (Rouse et al., 2020; Warburton et al., 2020). Findings from the two studies identified three to five need profiles (*e*.*g*., high satisfaction–low frustration profile, low satisfaction–very high frustration profile). While the findings, based on the domains of physical education and sport, provide support to the asymmetrical proposition, it is only partially supported in the work context given the presence of high competence satisfaction–high frustration profile. Therefore, future studies are warranted to investigate this proposition in the work context.

Identification of different need profiles has implications on the understanding of human functioning. For example, Rouse et al. (2020) found that firefighters from more adaptive need profiles (*e*.*g*., high satisfaction–low frustration profile) experienced fewer symptoms of anxiety, depression, and stress than those from less adaptive profiles (*e*.*g*., low satisfaction–very high frustration profile). Similarly, athletes from more adaptive profiles reported higher levels of well-being and enjoyment in sport training and lower levels of burnout than their counterparts from less adaptive profiles (Warburton et al., 2020). The findings unveiled some relevance of human functioning through experiencing varying combinations of need satisfaction and need frustration. In extension of the existing literature, we examined several need-relevant correlates, which include mindfulness, physical literacy, PA enjoyment, and PA. In addition to the theoretical relevance to the three basic psychological needs, these correlates are also important determinants of physical and psychological health (Raedeke, 2007; Cairney et al., 2019; Kee et al., 2019).

Mindfulness, a dispositional and trainable quality, refers to “paying attention in a particular way: on purpose, in the present moment, and non-judgmentally” (Kabat-Zinn, 2001). Mindfulness is believed to facilitate need satisfaction and decrease need frustration (Schultz et al., 2015). For example, in a PA setting, the receptive and non-judgmental awareness that characterizes mindfulness would help individuals to act in a way that is in line with their inner-value (autonomy), regulate attention and emotions to cope with physical challenges (competence), and attend to social interactions (relatedness). Indeed results derived from the variable-centered approach have indicated positive associations between mindfulness and need satisfaction as well as a negative relationship between mindfulness and need frustration across different contexts (Campbell et al., 2015; Schultz et al., 2015; Li et al., 2019a,b), yet none of the earlier research has examined the links between mindfulness and need satisfaction/frustration through a person-centered approach.

Physical literacy is defined as “a disposition acquired by individuals encompassing the motivation, confidence, physical competence, knowledge, and understanding that establishes purposeful physical pursuits as an integral element of their lifestyle” (Whitehead, 2013). According to the tenet of BPNT, experience of need satisfaction will lead to positive development and functioning, while frustration of basic psychological needs will result in negative outcomes (Deci and Ryan, 2000). In line with this tenet, need satisfaction in physical education was found to positively predict physical literacy in university students (Wang et al., 2020). Similarly, evidence has shown that satisfaction of basic psychological needs is positively related to positive outcomes such as intrinsic motivation, positive affect, enjoyment, and PA across health, physical education, and PA contexts, whereas need frustration is a negative predictor (Gunnell et al., 2013; Huhtiniemi et al., 2019; Vasconcellos et al., 2019). It is worthy to note that the empirical evidence from these studies were mainly based on the variable-centered approach (Vansteenkiste et al., 2020). To our best knowledge, no research examining how the need profiles would relate to varying levels of PA enjoyment and PA has been conducted.

As an extension of earlier research, the current cross-sectional study was undertaken to examine the combined associations between need satisfaction and need frustration (*i*.*e*., need profiles) and their relations with theoretically relevant correlates including mindfulness, physical literacy, PA enjoyment, and PA in school students. By employing a person-centered analytic approach while basing on the findings from previous research (Rouse et al., 2020; Warburton et al., 2020), we anticipated that at least two need profiles characterized by differences in need satisfaction and need frustration scores would emerge (*e*.*g*., high need satisfaction–low need frustration and low need satisfaction–high need frustration). We, however, did not expect a high need satisfaction and need frustration profile given that they are suggested to be asymmetrical (Vansteenkiste and Ryan, 2013). Finally, we expected that more adaptive need profiles would have a greater level of positive correlates (*i*.*e*., mindfulness, physical literacy, PA enjoyment, light PA, and moderate-to-vigorous PA) and a lower level of sedentary time as compared to less adaptive profiles (Rouse et al., 2020; Warburton et al., 2020).

## Methods

### Participants

To be eligible for this cross-sectional study, the participants must be fulltime students studying at a public school in Singapore. A sample of 844 school students were recruited from 19 primary schools (*n* = 384) and 20 secondary schools (*n* = 460). There were more boys (*n* = 448, 53.1%) than girls, and there was no gender difference across school levels [χ^2^(1) = 0.33, *p* = 0.56]. The participants had a mean age of 12.45 years (SD = 1.99; range = 9–17).

### Measures

We used five standardized scales to measure predictors and outcomes of interest. The predictors were six types of need satisfaction and need frustration. The outcome variables were mindfulness, physical literacy, PA enjoyment, sedentary time, light PA time, and moderate-to-vigorous PA time.

#### Need Satisfaction and Frustration

We adopted the Basic Psychological Needs Satisfaction and Frustration Scale (Chen et al., 2015) to measure the participants’ need satisfaction and need frustration in PA participation. The scale has six four-item subscales, with each subscale measuring one type of need satisfaction and frustration (*e*.*g*., “I feel confident that I can do things well”). The participants were asked to report their need experiences in PA participation and rate the items using a seven-point scale (1 = not true at all, 7 = completely true). We computed the six subscale scores for further analysis. A higher score would indicate a higher level of need satisfaction and need frustration.

#### Mindfulness

We employed the Child and Adolescent Mindfulness Measure (Greco et al., 2011) to assess the participants’ dispositional mindfulness level. The scale consists of 10 items (*e*.*g*., “At school, I walk from class to class without noticing what I’m doing”). The participants provided responses on a five-point scale (0 = never true, 4 = always true). A total mean scale score was computed, and a higher scale score would suggest a greater level of mindfulness.

#### Physical Literacy

We utilized the Perceived Physical Literacy Instrument (Sum et al., 2018) to measure the participants’ physical literacy. The participants used a five-point scale (1 = strongly disagree, 5 = strongly agree) to provide responses on the nine scale items (*e*.*g*., “I am physically fit, in accordance with my age”). A mean scale score was calculated. A higher mean scale score would suggest a greater level of physical literacy.

#### Physical Activity Enjoyment

We borrowed four scale items from the PA Enjoyment Scale (Raedeke, 2007) to measure the participants’ PA enjoyment. The participants were asked “How do you feel recently about the physical exercise you have been doing?” Four seven-point semantic scale items were used for responses (*e*.*g*., from “I enjoy it” to “I hate it”). A mean scale score was computed, where higher scale scores would represent greater levels of enjoyment in PA participation.

#### Physical Activity

We used the International PA Questionnaire-Short Form (Craig et al., 2003) to assess the participants’ subjective PA level across a week. This nine-item scale records four intensity levels of PA: vigorous PA, moderate PA, light PA, and sedentary time. According to the data analysis guideline^1^, sedentary time, light PA time, and moderate-to-vigorous PA time were computed and represented as total minutes per week.

### Data Collection

Recruitment of potential participants and data collection took place from March to December 2019. Upon receiving ethics approval from Nanyang Technological University (ID: IRB-2018-12-009) and the Ministry of Education (ID: 39926), an invitation email was sent to 40 school principals to participate in this survey. Of those invited, 39 school principals agreed to invite their students to participate in this survey. After obtaining the written informed consent forms from 1,066 participants and their parents/guardians, the anonymous survey forms were administered to the participants in a quiet sports hall or classroom. The participants were encouraged to give honest responses. A total of 844 participants completed the survey and were included in the analysis.

### Data Analysis

We used the following approaches to clean our data. We identified missing data points in some of the scale items (up to 1.9% in each item) and replaced them using expectation–maximization algorithm. We recoded univariate outliers to the nearest acceptable values (*Z* < 3.29). We did not identify any extreme multivariate outliers based on the results of Mahalanobis distance (*p* < 0.001; 25). Following the data cleaning process, we conducted a series of confirmatory factor analyses to examine the factorial validity of the psychological measures used. We applied the method of maximum likelihood estimation with robust standard errors for correcting bias induced by multivariate non-normality (Satorra and Bentler, 2010). We used comparative fit index (CFI), Tucker–Lewis index (TLI), root mean square error of approximation (RMSEA), and standardized root mean square residual (SRMR) to evaluate model fit. According to Hu and Bentler (1998), values for CFI/TLI greater than 0.90, values for RMSEA less than 0.06, and values for SRMR less than 0.08 are deemed acceptable. Furthermore, we computed internal reliability (Cronbach’s α), descriptive statistics (*M* and SD), and bivariate correlations for the major study variables.

Lastly, we used a person-centered approach (*i*.*e*., LPA) to identify the optimal number of need profiles. We specified and estimated models with one to seven profiles using the six need satisfaction and need frustration subscale scores (*i*.*e*., six need indicators). We used several fit measures to identify the best model: Akaike’s Information Criterion (AIC), Bayesian Information Criterion (BIC), Sample-Adjusted BIC (SABIC), Lo–Medell–Rubin Adjusted Likelihood Ratio Test (LALRT), and the bootstrap likelihood ratio test (BLRT). Lower values of AIC, BIC, and SABIC would suggest a better model fit. A statistically significant finding of LALRT and BLRT would suggest that the model (*k* + 1 profile) is preferred to the model with one less profile (*k* profile). It is worthy to note that the BLRT result may always be statistically significant (Masyn, 2013). In addition to using these fit indices, we also considered values of entropy and average posterior probability, number of cases in a profile, and interpretability of the model for determining the optimal number of need profiles. Values for entropy higher than 0.60 and values for average posterior probability greater than 0.80 are considered acceptable (Nagin, 1999). A model containing a profile with less than 5% of the total sample was discarded (Masyn, 2013). To increase the interpretability of the six need indicators, we calculated their *z*-scores. Values of ±0.49 SD were classified as average, ±0.5 to 0.99 SD as high/low, and ±1 SD as very high/low (Rouse et al., 2020).

Following the identification of the optimal number of need profiles, we conducted a series of Wald chi-square test to examine whether the identified need profiles were related to theoretically relevant correlates, including mindfulness, physical literacy, PA enjoyment, sedentary time, light PA time, and moderate-to-vigorous PA time (Asparouhov and Muthén, 2014). We cleaned our data and conducted preliminary analyses using IBM SPSS Statistics 25 (IBM, Armonk, NY, United States). We conducted the confirmatory factor analyses and LPA using *M*plus 8 (Muthén and Muthén, 1998–2017).

## Results

### Measurement Model

The results of confirmatory factor analysis supported the factorial validity of the psychological measures used. Specifically, the unidimensional model of the Child and Adolescent Mindfulness Measure had an adequate model fit: χ^2^(24) = 200.37, CFI = 0.927, TLI = 0.897, RMSEA = 0.079 [90% CI (0.069, 0.090)], SRMR = 0.052. The adequacy of the first-order six-factor measurement model of the Basic Psychological Needs Satisfaction and Frustration Scale was supported: χ^2^(237) = 600.36, CFI = 0.949, TLI = 0.940, RMSEA = 0.043 [90% CI (0.038, 0.047)], SRMR = 0.044. The second-order three-factor measurement model of the Perceived Physical Literacy Instrument demonstrated an adequate model fit: χ^2^(24) = 72.01, CFI = 0.984, TLI = 0.976, RMSEA = 0.049 [90% CI (0.036, 0.062)], SRMR = 0.024. Finally, the data fit the one-factor measurement model of PA Enjoyment Scale well: χ^2^(1) = 4.85, CFI = 0.999, TLI = 0.992, RMSEA = 0.0768 [90% CI (0.018, 0.133)], SRMR = 0.003.

### Descriptive Statistics

Table 1 shows the internal reliability estimates, means, standard deviations, and bivariate correlations for the major study variables. The scales/subscales demonstrated adequate to excellent internal reliability with the current sample (α = 0.70–0.95). The participants reported a moderate level of need satisfaction and physical literacy, a slightly below average level of need frustration, an average level of mindfulness, and a relatively high level of PA enjoyment. Over a 1-week period, the participants reported 3,180.71 min of sedentary time, participated in 767.95 min of light PA, and engaged in 449.32 min of moderate-to-vigorous PA. In line with our expectation, need satisfaction subscale scores were positively related to physical literacy, PA enjoyment, and moderate-to-vigorous PA. However, there was a negative and weak association between autonomy satisfaction and mindfulness. The need frustration scale scores, as expected, were negatively associated with mindfulness.

**TABLE 1:** Descriptive statistics and zero-order correlations of the study variables.

	α	M (SD)	Scale	1	2	3	4	5	6	7	8	9	10	11
Autonomy satisfaction	0.71	3.67 (0.71)	1–7	–										
Competence satisfaction	0.85	3.67 (0.80)	1–7	0.63**		–								
Relatedness satisfaction	0.84	3.93 (0.77)	1–7	0.53**	0.50**	–								
Autonomy frustration	0.70	2.72 (0.82)	1–7	–0.06	−0.10**	−0.09**	–							
Competence frustration	0.80	2.65 (0.94)	1–7	−0.09*	−0.30**	−0.19**	0.61**	–						
Relatedness frustration	0.81	2.29 (0.93)	1–7	–0.03	−0.07*	−0.28**	0.61**	0.64**	–					
Mindfulness	0.85	2.03 (0.80)	0–4	−0.14**	–0.001	–0.03	−0.31**	−0.44**	−0.34**	–				
Physical literacy	0.89	3.74 (0.71)	1–5	0.45**	0.64**	0.38**	–0.01	−0.18**	–0.02	−0.12**	–			
PA enjoyment	0.95	5.39 (0.71)	1–7	0.30**	0.34**	0.21**	−0.09**	−0.18**	–0.06	–0.04	0.42**	–		
Sedentary time	–	3,180.71 (1523.32)	–	−0.11**	−0.12**	–0.06	–0.01	0.05	–0.02	0.02	−0.13**	−0.12**	–	
Light PA	–	767.95 (925.31)	–	0.001	0.02	0.06	–0.001	0.02	0.04	−0.07*	0.06	0.09*	0.07*	–
Moderate-to-vigorous PA	–	449.34 (421.38)	–	0.18**	0.31**	0.15**	0.01	−0.09**	0.01	–0.05	0.41**	0.27**	−0.09**	0.18**

PA, physical activity. **p < 0.01; *p < 0.05.

### Need Profiles

Table 2 shows the model parameters of LPA. The six- and seven-profile models that contained a group size with less than 5% of the total sample were subsequently dropped. Although all the results of BLRT were significant, the result of BALRT only reached significance in the two-, three-, and four-profile models. The four-profile model had relatively lower values of AIC, BIC, and SBIC than the two- and three-profile models. Taking into further consideration factors of interpretability and its acceptable entropy and average posterior probability values, the four-profile model was finally selected.

**TABLE 2:** Summary of model parameters for latent profile analysis.

*k*	AIC	BIC	SABIC	LALRT *p*-value	BLRT *p*-value	Group size ≤ 5%	Entropy	APP
1	12,431.11	12,487.96	12,449.86	–	–	0	–	–
2	11,681.20	11,771.23	11,710.89	0.04	<0.001	0	0.72	0.92
3	10,971.36	11,094.56	11,011.99	<0.001	<0.001	0	0.83	0.92
4	10,789.97	10,946.33	10,841.53	0.01	<0.001	0	0.78	0.88
5	10,671.48	10,861.00	10,733.98	0.61	<0.001	0	0.75	0.85
6	10,589.06	10,811.76	10,662.50	0.21	<0.001	1	0.78	0.86
7	10,505.45	10,761.31	10,589.82	0.17	<0.001	1	0.79	0.87

**k*, number of profiles; AIC, Akaike’s Information Criterion; BIC, Bayesian Information Criterion; SABIC, sample-size-adjusted BIC; LALRT, Lo–Medell–Rubin adjusted likelihood ratio test; BLRT, bootstrapped likelihood ratio test; APP, average posterior probability.*

**FIGURE 1:**
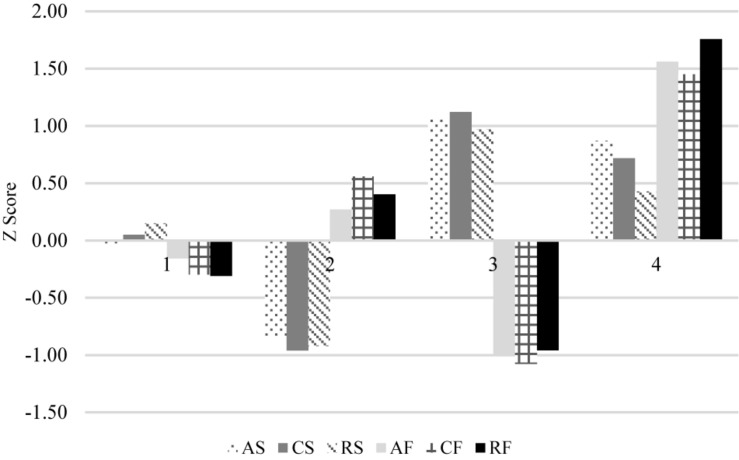
Characteristics of the four-profile model based on the six need indicators. Profile 1 = average satisfaction and frustration (*n* = 364, 44.1%), profile 2 = low satisfaction, above average frustration (*n* = 251, 29.7%), profile 3 = very high satisfaction, very low frustration (*n* = 144, 17.1%), profile 4 = high satisfaction, very high frustration (*n* = 85, 10.1%). AS, autonomy satisfaction, CS, competence satisfaction, RS, relatedness satisfaction, AF, autonomy frustration, CF, competence frustration, RF, relatedness frustration.

Figure 1 presents the characteristics of the four-profile model. Profile 3 was the most adaptive profile and was described as “very high satisfaction, very low frustration” (*n* = 144, 17.1%). In contrast, profile 2 was the least adaptive profile with “low satisfaction, above average frustration” (*n* = 251, 29.7%). Profile 1 was described as “average satisfaction and frustration” (*n* = 364, 44.1%). Finally, profile 4 was described as “high satisfaction, very high frustration” (*n* = 85, 10.1%).

### Correlates of Need Profiles

Table 3 summarizes the descriptive statistics for need satisfaction or need frustration and correlates. The four identified profiles differed significantly in mindfulness, physical literacy, PA enjoyment, and moderate-to-vigorous PA (overall Wald χ^2^ = 5.67–69.03, *p* < 0.05). The results of pairwise comparisons confirmed that profile 3 was the most adaptive profile, which had the highest levels of mindfulness, physical literacy, PA enjoyment, and moderate-to-vigorous PA. Profile 2 was the most maladaptive profile, which reported the lowest levels in three out of four significant correlates. Unexpectedly, there were no significant differences in sedentary time (overall Wald χ^2^ = 1.83, *p* = 0.18) and light PA (overall Wald χ^2^ = 0.05, *p* = 0.82) across the four identified profiles.

**TABLE 3:** Descriptive statistics for need satisfaction/frustration and correlates.

Variables	Profile 1 Average satisfaction and frustration		Profile 2 Low satisfaction, above average frustration		Profile 3 Very high satisfaction, very low frustration		Profile 4 High satisfaction, very high frustration		Pairwise comparisons between profiles^a^
					
	*M* (SD)	*Z*	*M* (SD)	*Z*	*M* (SD)	*Z*	*M* (SD)	*Z*	
**Indicators**									
Autonomy satisfaction	3.64 (0.52)	–0.04	3.07 (0.54)	–0.85	4.43 (0.44)	1.07	4.30 (0.50)	0.87	–
Competence satisfaction	3.71 (0.57)	0.05	2.90 (0.55)	–0.96	4.57 (0.42)	1.12	4.24 (0.55)	0.72	–
Relatedness satisfaction	4.04 (0.55)	0.15	3.22 (0.67)	–0.92	4.67 (0.44)	0.97	4.25 (0.55)	0.43	–
Autonomy frustration	2.60 (0.58)	–0.16	2.95 (0.62)	0.27	1.91 (0.63)	–1.00	4.00 (0.64)	1.56	–
Competence frustration	2.37 (0.63)	–0.30	3.17 (0.71)	0.56	1.63 (0.52)	–1.08	4.01 (0.64)	1.45	–
Relatedness frustration	2.01 (0.60)	–0.31	2.67 (0.68)	0.40	1.40 (0.50)	–0.96	3.93 (0.69)	1.76	–
**Correlates**									
Mindfulness	2.14 (0.74)	–	1.94 (0.74)	–	2.30 (0.78)	–	1.42 (0.95)	–	3 = 4 > 1 > 2
Physical literacy	3.77 (0.56)	–	3.24 (0.68)	–	4.24 (0.51)	–	4.24 (0.64)	–	3 > 1 > 2 > 4
PA enjoyment	5.45 (0.64)	–	4.74 (1.43)	–	6.19 (1.32)	–	5.74 (1.63)	–	3 > 4^b^ = 1 > 2
Sedentary time	3,153.38 (1411.11)	–	3,418.43 (1683.15)	–	3,064.74 (1441.19)	–	2,792.26 (1538.76)	–	–^c^
Light PA	737.97 (836.70)	–	783.18 (1002.34)	–	795.09 (990.83)	–	805.35 (947.95)	–	–^c^
Moderate-to-vigorous PA	444.99 (421.45)	–	327.53 (372.38)	–	607.57 (445.26)	–	559.55 (405.62)	–	3 = 4 > 1 > 2

*PA, physical activity. ^*a*^All the differences are significant at the level of *p* < 0.05 (two-tailed) if not otherwise specified. ^*b*^There is a trend that profile 3 has a higher level of PA enjoyment than that of profile 4 (*p* = 0.05). ^*c*^The result of the overall Wald test is not significant.*

## Discussion

In extension of previous research, we examined the need profiles and their associations with theoretically relevant correlates in Singapore school students. Our findings revealed four distinct need profiles in the context of PA participation, including profile 1–average satisfaction and frustration; profile 2–low satisfaction, above average frustration; profile 3–very high satisfaction, very low frustration; and profile 4–high satisfaction, very high frustration. A more adaptive profile was characterized by a stronger presence of need satisfaction over need frustration (*e*.*g*., profile 3), whereas a less adaptive profile was characterized by need frustration prevailing over need satisfaction (*e*.*g*., profile 2). Furthermore, the participants from a more adaptive need profile generally reported a greater level of positive correlates than those from a less adaptive one.

In line with our expectations, more than two (*i*.*e*., four) distinct need profiles emerged from LPA. The finding is similar to previous studies conducted in the contexts of work, physical education, and sport (Rouse et al., 2020; Warburton et al., 2020). In these studies, three to five need profiles were identified. The contextual differences may have contributed to the varied number of profiles that emerged across these studies. The emergence of several typical need profiles (*e*.*g*., high satisfaction–low frustration profile, low satisfaction–high frustration profile), however, suggests that there is a degree of stability and generalizability of the need profiles across the contexts. While these typical need profiles provide some support for the asymmetrical hypothesis, there is still limited empirical evidence to fully support this proposition. For example, profile 4 (high satisfaction, very high frustration) was with both need satisfaction and need frustration scores above average, failing to support the asymmetrical hypothesis. The study of Rouse et al. (2020) also found a similar issue (*e*.*g*., very high competence satisfaction, high frustration). Hence, more studies are needed to test the asymmetrical hypothesis given the limited empirical evidence for it.

Previous research has shown that different need profiles were uniquely associated with varied levels of correlates such as anxiety, depression, motivation, and life satisfaction (Vansteenkiste et al., 2020). In line with and as an extension to previous research (Vansteenkiste and Ryan, 2013; Rouse et al., 2020; Vansteenkiste et al., 2020; Warburton et al., 2020), each of the four identified need profiles in the present study was found to have unique associations with mindfulness, physical literacy, PA enjoyment, and moderate-to-vigorous PA. However, the identified four need profiles showed no difference in light PA and sedentary behavior. Although mounting evidence shows that moderate-to-vigorous PA leads to multiple health-related outcomes, there is still limited evidence supporting the association between light PA/sedentary behavior and its outcomes (Chaput et al., 2020). Thus, it could be possible that light PA and sedentary behavior are not a sensitive correlate of need profile in comparison to moderate-to-vigorous PA. More studies are warranted to confirm this possibility.

The present findings showed that profile 3 (very high satisfaction, very low frustration) was the most adaptive, which had the highest level of mindfulness, physical literacy, PA enjoyment, and moderate-to-vigorous PA among the four identified profiles. In contrast, profile 2 (low satisfaction, above average frustration) was the least adaptive profile, as it had the lowest levels of mindfulness, PA enjoyment, and moderate-to-vigorous PA. Interestingly, although profile 4 (high satisfaction, very high frustration) had the highest need frustration score, it is still more adaptive than profile 1 (average satisfaction and frustration) and profile 2 (low satisfaction, above average frustration). Further inspection of the need scores across these three profiles indicated that profile 4 (high satisfaction, very high frustration) had the highest need satisfaction score albeit with the highest need frustration score. This would suggest that a high need satisfaction score might be necessary for optimal human functioning. This finding further suggests that high need satisfaction could protect or offset the negative effects of very high need frustration on human functioning. According to Warburton et al. (2020), experiences of high need frustration were less detrimental to enjoyment and well-being when athletes experienced moderate levels of need satisfaction. Thus, experiences of need frustration may not necessarily be maladaptive, and it is important to simultaneously examine the interplay between need satisfaction and need frustration.

### Practical Implications

There are significant implications that we can recommend based on the findings and discussion outlined above. The use of the person-centered approach can help practitioners identify student groups that are at risk of dysfunction. In our study, about one-third (29.7%) of the participants was from profile 2 (low satisfaction, above average frustration), the least adaptive profile. To facilitate optimal student functioning, it is recommended to alter the PA environment to reduce students’ experiences of need frustration. Furthermore, given the protective role of need satisfaction in alleviating the negative effects of need frustration on student functioning, it is more important to enhance the students’ need satisfaction. Approaches such as use of non-controlling language that conveys freedom of choice, acknowledging one’s perspective, and assisting in identifying realistic goals can be used to prompt need satisfaction (Teixeira et al., 2020).

### Limitations and Future Research

Despite contributing to the BPNT literature and providing practical implications, the present study is subject to some limitations. Firstly, we examined need profiles in the context of PA participation with Singapore school students; generalization of our study findings is therefore limited. Further studies should examine the different life domains, countries, and school levels to collaborate and extend the present study findings. Secondly, the use of a cross-sectional survey design prevents us from inferring casual associations between need profiles and their correlates. Future research can use a prospective survey design to examine the predictability of need profiles on the same and additional correlates that are important for human functioning. By employing this approach, we can also examine how need satisfaction and need frustration associate and influence each other. Future research can even use an experimental approach to examine the causal associations. Finally, we exclusively relied on using self-report measures that would potentially bias the results of the study. It is therefore useful for future research to employ objective measures to assess some of the study variables (*e*.*g*., use heart rate monitors or accelerometers to measure PA).

## Conclusion

In summary, our person-centered analyses revealed four combinations of need satisfaction and need frustration in a PA setting (*i*.*e*., profile 1–average satisfaction and frustration; profile 2–low satisfaction, above average frustration; profile 3–very high satisfaction, very low frustration; and profile 4–high satisfaction, very high frustration). The identified combinations (need profiles) were strongly related to several correlates, including mindfulness, physical literacy, physical activity enjoyment, and moderate-to-vigorous physical activity. Although the asymmetrical relationship between need satisfaction and need frustration was not fully supported, experiences of need satisfaction countered the negative effects of need frustration on the correlates. These findings enhance our understanding of psychological need experiences. The findings also highlight the need for further investigation of the combined associations between need satisfaction and need frustration, which could provide unique and complementary explanations to human functioning.

## Data Availability Statement

The original contributions presented in the study are included in the article/supplementary material, further inquiries can be directed to the corresponding author.

## Ethics Statement

The studies involving human participants were reviewed and approved by Nanyang Technological University. Written informed consent to participate in this study was provided by the participants’ legal guardian/next of kin.

## Author Contributions

All authors were involved in manuscript preparation and approved the final version for submission.

## Conflict of Interest

The authors declare that the research was conducted in the absence of any commercial or financial relationships that could be construed as a potential conflict of interest.

## Footnotes

1 www.ipaq.ki.se

## References

[B1] Asparouhov T. Muthén B. (2014). Auxiliary variables in mixture modeling: three-step approach using Mplus. *Struct. Equ. Modeling* 21 329–341. 10.1080/10705511.2014.915181

[B2] Bartholomew K. J. Ntoumanis N. Ryan R. M. Bosch J. A. Thogersen-Ntoumani C. (2011). Self-determination theory and diminished functioning: the role of interpersonal control and psychological need thwarting. *Pers. Soc. Psychol. Bull.* 37 1459–1473. 10.1177/0146167211413125 21700794

[B3] Cairney J. Dudley D. Kwan M. Bulten R. Kriellaars D. (2019). Physical literacy, physical activity and health: toward an evidence-informed conceptual model. *Sports Med.* 49 371–383. 10.1007/s40279-019-01063-3 30747375

[B4] Campbell R. Vansteenkiste M. Delesie L. M. Mariman A. N. Soenens B. Tobback E. (2015). Examining the role of psychological need satisfaction in sleep: a self-determination theory perspective. *Pers. Individ. Differ.* 77 199–204. 10.1016/j.paid.2015.01.003

[B5] Chaput J. Willumsen J. Bull F. Chou R. Firth J. Jago R. (2020). 2020 WHO guidelines on physical activity and sedentary behaviour for children and adolescents aged 5-17 years: summary of the evidence. *Int. J. Behav. Nutr. Phys. Act.* 17 2–9. 10.1186/s12966-020-01037-z 33239009 PMC7691077

[B6] Chen B. W. Vansteenkiste M. Beyers W. Boone L. Deci E. L. van der Kaap-Deeder J. (2015). Basic psychological need satisfaction, need frustration, and need strength across four cultures. *Motiv. Emot.* 39 216–236. 10.1007/s11031-014-9450-1

[B7] Craig C. L. Marshall A. L. Sjöström M. Bauman A. E. Booth M. L. Ainsworth B. E. (2003). International physical activity questionnaire: 12-country reliability and validity. *Med. Sci. Sports Exerc.* 35 1381–1395. 10.1249/01.MSS.0000078924.61453.FB 12900694

[B8] De Francisco C. Parra F. J. Arce C. Vílchez M. D. P. (2018). Preliminary empirical validation of the “Basic needs satisfaction in sport scale” with a sample of Spanish athletes. *Front. Psychol.* 9:1057. 10.3389/fpsyg.2018.01057 29997550 PMC6030354

[B9] Deci E. L. Ryan R. M. (2000). The “what” and the “why” of goal pursuits: human needs and the self-determination of behavior. *Psychol. Inquiry* 11 227–268. 10.1207/S15327965PLI1104_01

[B10] Greco L. A. Baer R. A. Smith G. T. (2011). Assessing mindfulness in children and adolescents: development and validation of the Child and Adolescent Mindfulness Measure (CAMM). *Psychol. Assess.* 23 606–614. 10.1037/a0022819 21480722

[B11] Gunnell K. E. Crocker P. R. Wilson P. M. Mack D. E. Zumbo B. D. (2013). Psychological need satisfaction and thwarting: a test of basic psychological needs theory in physical activity contexts. *Psychol. Sport Exerc.* 14 599–607. 10.1016/j.psychsport.2013.03.007

[B12] Howard M. C. Hoffman M. E. (2018). Variable-centered, person-centered, and person-specific approaches: where theory meets the method. *Organ. Res. Methods* 21 846–876. 10.1177/1094428117744021

[B13] Hu L. T. Bentler P. M. (1998). Fit indices in covariance structure modeling: sensitivity to underparameterized model misspecification. *Psychol. Methods* 3 424–453. 10.1037/1082-989X.3.4.424

[B14] Huhtiniemi M. Sääkslahti A. Watt A. Jaakkola T. (2019). Associations among basic psychological needs, motivation and enjoyment within Finnish physical education students. *J. Sports Sci. Med.* 18 239–247. 31191093 PMC6544006

[B15] Kabat-Zinn J. (2001). *Mindfulness Meditation for Everyday Life.* London: Piatkus Books.

[B16] Kee Y. H. Li C. Kong L. C. Tang C. J. Chuang K. L. (2019). Scoping review of mindfulness research: a topic modelling approach. *Mindfulness* 10 1474–1488. 10.1007/s12671-019-01136-4

[B17] Li C. Kee Y. H. Kong L. C. Zou L. Ng K. L. Li H. (2019a). Autonomy-supportive teaching and basic psychological need satisfaction among school students: the role of mindfulness. *Int. J. Environ. Res. Public Health* 16:2599. 10.3390/ijerph16142599 31330926 PMC6679142

[B18] Li C. Wong N. K. Sum R. K. Yu C. W. (2019b). Preservice teachers’ mindfulness and attitudes toward students with autism spectrum disorder: the role of basic psychological needs satisfaction. *Adapt. Phys. Activ. Q.* 36 150–163. 10.1123/apaq.2018-0044 30554521

[B19] Masyn K. E. (2013). “Latent class analysis and finite mixture modeling,” in *The Oxford Handbook of Quantitative Methods*, ed. Little T. (New York, NY: Oxford University Press), 551–611.

[B20] Muthén L. K. Muthén B. O. (1998–2017). *Mplus User’s Guide.* Los Angeles, CA: MuthénMuthén.

[B21] Nagin D. S. (1999). Analyzing developmental trajectories: a semiparametric, group-based approach. *Psychol. Methods* 4 139–157. 10.1037/1082-989X.4.2.139 11285809

[B22] Raedeke T. D. (2007). The relationship between enjoyment and affective responses to exercise. *J. Appl. Sport Psychol.* 19 105–115.

[B23] Rouse P. C. Turner P. J. Siddall A. G. Schmid J. Standage M. Bilzon J. L. (2020). The interplay between psychological need satisfaction and psychological need frustration within a work context: a variable and person-oriented approach. *Motiv. Emot.* 44 175–189. 10.1080/10413200601113638

[B24] Satorra A. Bentler P. M. (2010). Ensuring positiveness of the scaled difference chi-square test statistic. *Psychometrika* 75 243–248. 10.1007/s11336-009-9135-y 20640194 PMC2905175

[B25] Schultz P. P. Ryan R. M. Niemiec C. P. Legate N. Williams G. C. (2015). Mindfulness, work climate, and psychological need satisfaction in employee well-being. *Mindfulness* 6 971–985. 10.1007/s12671-014-0338-7

[B26] Sum R. K. Cheng C. F. Wallhead T. Kuo C. C. Wang F. J. Choi S. M. (2018). Perceived physical literacy instrument for adolescents: a further validation of PPLI. *J. Exerc. Sci. Fit.* 16 26–31. 10.1016/j.jesf.2018.03.002 30662489 PMC6323161

[B27] Teixeira P. J. Marques M. M. Silva M. N. Brunet J. Duda J. L. Haerens L. (2020). Classification of techniques used in self-determination theory-based interventions in health contexts: an expert consensus study. *Motiv. Sci.* 6 438–455. 10.1037/mot0000172

[B28] Vansteenkiste M. Ryan R. M. (2013). On psychological growth and vulnerability: basic psychological need satisfaction and need frustration as an unifying principle. *J. Psychother. Integr.* 23 263–280. 10.1037/a0032359

[B29] Vansteenkiste M. Ryan R. M. Soenens B. (2020). Basic psychological need theory: advancements, critical themes, and future directions. *Motiv. Emot.* 44 1–31. 10.1007/s11031-019-09818-1

[B30] Vasconcellos D. Parker P. D. Hilland T. Cinelli R. Owen K. B. Kapsal N. (2019). Self-determination theory applied to physical education: a systematic review and meta-analysis. *J. Educ. Psychol.* 112 1444–1469. 10.1037/edu0000420

[B31] Wang F. J. Cheng C. F. Chen M. Y. Sum K. W. R. (2020). Temporal precedence of physical literacy and basic psychological needs satisfaction: a cross-lagged longitudinal analysis of university students. *Int. J. Environ. Res. Public Health* 17:4615. 10.3390/ijerph17124615 32604980 PMC7345862

[B32] Warburton V. E. Wang J. C. Bartholomew K. J. Tuff R. L. Bishop K. C. (2020). Need satisfaction and need frustration as distinct and potentially co-occurring constructs: need profiles examined in physical education and sport. *Motiv. Emot.* 44 54–66. 10.1007/s11031-019-09798-2

[B33] Whitehead M. (2013). “What is physical literacy and how does it impact on physical education?,” in *Debates in Physical Education*, eds Capel S. Whitehead M. (London: Routledge), 37–52.

